# Calcium-binding site in AA10 LPMO from *Vibrio cholerae* suggests modulating effects during environmental survival and infection

**DOI:** 10.1017/qrd.2024.14

**Published:** 2024-12-26

**Authors:** Mateu Montserrat-Canals, Kaare Bjerregaard-Andersen, Henrik Vinther Sørensen, Eirik Kommedal, Gabriele Cordara, Gustav Vaaje-Kolstad, Ute Krengel

**Affiliations:** 1 Centre for Molecular Medicine Norway, University of Oslo, NO-0318 Oslo, Norway; 2 Department of Chemistry, University of Oslo, NO-0315 Oslo, Norway; 3 Faculty of Chemistry, Biotechnology and Food Science, Norwegian University of Life Sciences (NMBU), NO-1433 Ås, Norway

**Keywords:** bacterial adhesin, biofilm formation, calcium, chitin, copper monooxygenase, GbpA, magnesium, metal-binding site, microbial pathogenesis, *Vibrio cholerae*, X-ray crystal structure

## Abstract

Despite major efforts toward its eradication, cholera remains a major health threat and economic burden in many low- and middle-income countries. Between outbreaks, the bacterium responsible for the disease, *Vibrio cholerae*, survives in aquatic environmental reservoirs, where it commonly forms biofilms, for example, on zooplankton. *N*-acetyl glucosamine-binding protein A (GbpA) is an adhesin that binds to the chitinaceous surface of zooplankton and breaks its dense crystalline packing thanks to its lytic polysaccharide monooxygenase (LPMO) activity, which provides *V. cholerae* with nutrients. In addition, GbpA is an important colonization factor associated with bacterial pathogenicity, allowing the binding to mucins in the host intestine. Here, we report the discovery of a cation-binding site in proximity of the GbpA active site, which allows Ca^2+^, Mg^2+^, or K^+^ binding close to its carbohydrate-binding surface. In addition to the X-ray crystal structures of cation-LPMO complexes (to 1.5 Å resolution), we explored how the presence of ions affects the stability and activity of the protein. Calcium and magnesium ions were found to bind to GbpA specifically, with calcium ions – abundant in natural sources of chitin – having the strongest effect on protein stability. When the cation-binding site was rendered non-functional, a decrease in activity was observed, highlighting the importance of the structural elements stabilized by calcium. Our findings suggest a cation-binding site specific to GbpA and related LPMOs that may fine-tune binding and activity for its substrates during environmental survival and host infection.

## Graphical abstract

**Figure:**
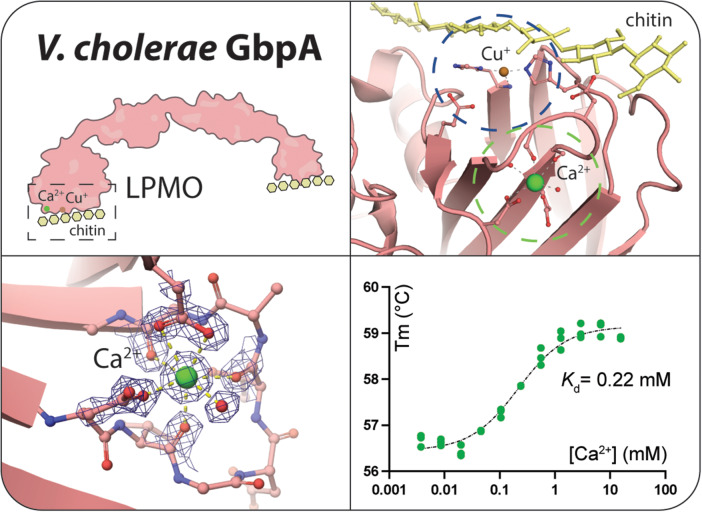

## Introduction

Cholera is an ancient and severe diarrheal disease (Kanungo et al., 2022). The current cholera pandemic is responsible for over 140.000 deaths annually in communities and countries where proper water sanitation is limited, especially in the wake of natural disasters and armed conflicts (Grant et al., 2021). The Gram-negative bacterium *Vibrio cholerae* is the pathogenic agent responsible for cholera.

**Figure 1.**
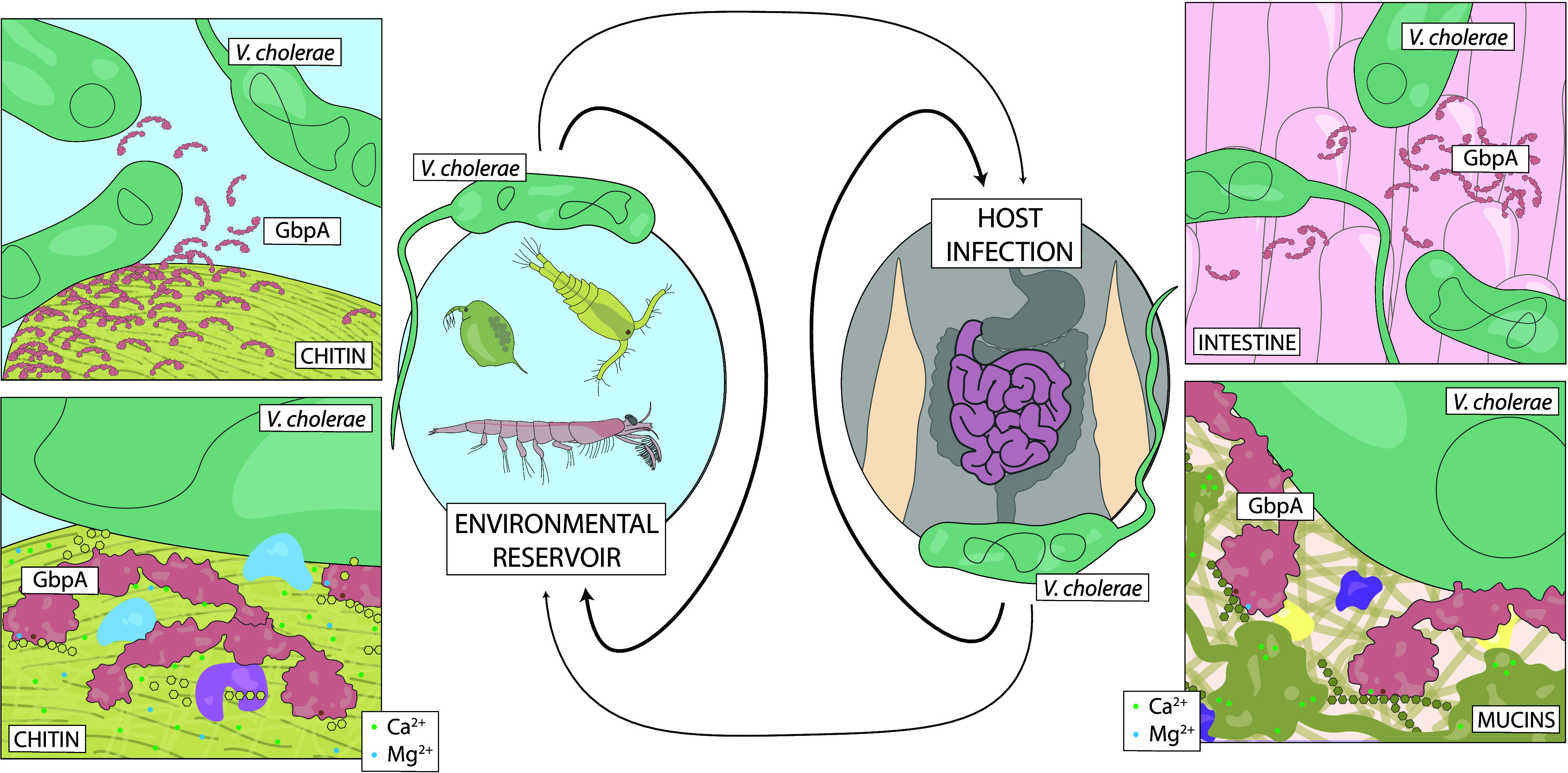
**Different niches of *V. cholerae* and functions of GbpA.** As a member of the marine microbiome, *V. cholerae* survives attached to biotic and abiotic surfaces. Of particular importance are the chitinous exoskeletons of marine crustaceans found in zooplankton, where *V. cholerae* forms microcolonies and uses the crystalline polysaccharide as a source of nutrients. To this process, GbpA is of particular importance, acting as a colonization factor by binding chitin and as part of the chitin utilization machinery, oxidatively degrading the polysaccharide chains, thereby allowing further processing by other enzymes. During pathogenesis and host infection, *V. cholerae* colonizes the intestine using colonization factors that mediate attachment. Here, GbpA also acts as a colonization factor by recognizing GlcNAc moieties present in the intestine, particularly from the highly glycosylated mucins. Crucially, different concentrations of calcium and magnesium ions are found in the different substrates of GbpA.

*V. cholerae* is a facultative pathogen found in aquatic environments worldwide. Traditionally assumed endemic to tropical regions, it is now regarded as a cosmopolitan species able to survive in a wide range of conditions (Lutz et al., 2013). As many other members of the *Vibrio* genus, *V. cholerae* can be found both as a free-living bacterium and attached to biotic and abiotic surfaces (Hood and Winter, 1997; Almagro-Moreno and Taylor, 2013; Lutz et al., 2013), where it often forms biofilms (Pruzzo et al., 2008; Yildiz and Visick, 2009). Among the multiple surfaces and organisms that act as environmental reservoirs of *V. cholerae*, chitin and its presence in the exoskeleton of planktonic crustaceans are of particular relevance (Figure 1). Attachment to chitin provides *V. cholerae* with an abundant and stable carbon and nitrogen source, allows lateral transmission of genetic mobile elements linked with pathogenicity (Meibom et al., 2005), and provides protection from predators (Pruzzo et al., 2008; Vezzulli et al., 2008). In addition to its role as a reservoir, chitin acts as a critical factor for cholera transmission (Colwell et al., 2003). Attachment to chitin is mediated by a range of colonization factors and adhesins (Pruzzo et al., 2008). A colonization factor of particular importance is *N*-acetyl glucosamine (GlcNAc)-binding protein A (GbpA) (Stauder et al., 2010), which is found in all *V. cholerae* strains (Stauder et al., 2012).

GbpA can bind the GlcNAc chains of chitin (Wong et al., 2012), and oxidatively degrades the polymers by means of its lytic polysaccharide monooxygenase (LPMO) activity (Loose et al., 2014). Discovered only recently (Vaaje-Kolstad et al., 2010), LPMOs are of interest for biomass conversion due to their ability to degrade recalcitrant polysaccharides (Srivastava et al., 2023). The oxidative activity of LPMOs depends on a copper ion bound by a histidine brace motif. Interestingly, bacterial LPMOs are often associated with pathogenicity (Frederiksen et al., 2013) – for instance, chitin-binding protein D, the tri-modular LPMO of *Pseudomonas aeruginosa*, is crucial for systemic infection (Askarian et al., 2021, 2023). In pathogenic *V. cholerae* strains, GbpA mediates binding to GlcNAc moieties in host intestinal mucins, making it an important virulence factor in addition to its role in environmental survival (Figure 1) (Kirn et al., 2005; Bhowmick et al., 2008). The structure of GbpA has been characterized previously: the crystal structure of the first three domains of the four-domain protein has been solved by X-ray crystallography, showing that domain 3 folds back on LPMO domain 1; whereas the solution structure of the full-length protein determined by small-angle X-ray scattering (SAXS) reveals an elongated conformation (Wong et al., 2012). The first and fourth domains are responsible for chitin binding, with only the first domain showing LPMO activity and additionally binding intestinal mucins (Wong et al., 2012). The second and third domains are likely responsible for interaction with still unidentified bacterial components (Wong et al., 2012).

Salinity and the concentrations of specific ions change significantly between the different niches that pathogenic *V. cholerae* colonize, for example, estuarine waters and the human intestine. Previous studies have investigated the optimal salinity for *V. cholerae* growth, survival, and attachment to chitin (Huq et al., 1984) as well as the effects of different salts on its attachment to inert surfaces (Hood and Winter, 1997). Moreover, the formation of *V. cholerae* biofilms has been shown to be negatively regulated by calcium (Bilecen and Yildiz, 2009). Apart from this, very little is known about how salinity and specific ions influence *V. cholerae* and its ability to colonize and survive on different surfaces, from the environmental reservoirs to the highly complex intestinal tracts of its hosts. Here, we report the discovery of a previously uncharacterized ion-binding site close to the active site of the GbpA LPMO domain, which affects protein stability and potentially fine-tunes its oxidative activity and binding to different substrates.

## Methods

### Expression constructs and mutagenesis

The *gbpA* gene (UniProt ID: Q9KLD5, residues 24–485) was codon-optimized and cloned into a pET-26b vector by GenScript® (Leiden, The Netherlands) between the restriction sites *Nco*I and *Xho*I, for expression in *Escherichia coli* BL21 (DE3). The gene was cloned from residue 24 – omitting the natural export signal – such that the PelB leader sequence in the vector would direct the protein to the periplasmic space. The signal peptide was subsequently removed by the expression host. The C-terminal His tag present in the vector was omitted by the inclusion of a stop codon at the end of the insert. The pET-26b vector contains a kanamycin resistance gene, which we exploited for selection in growth and expression phases. For the production of the LPMO domain of GbpA, the construct is equivalent to the full-length GbpA-containing residues 24–203.

Point mutations were introduced in the *gbpA* sequence using the NEB Q5 Site-Directed Mutagenesis Kit. Successful production of mutants was confirmed by sequencing the newly generated vectors.

### Protein production and purification

Two expression protocols were used for producing GbpA, one using rich and one using minimal media (Sørensen et al., 2023a). To produce protein for crystallization experiments, *E. coli* BL21 (DE3) cells were grown at 37 °C in LB medium containing 100 μg/mL ampicillin until OD_600_ of 0.6 was reached. The temperature was reduced to 20 °C and isopropyl-β-D-thiogalactopyranoside (IPTG) was added to a final concentration of 0.5 mM to induce expression. Cells were harvested after 20 h by centrifugation (8000 × *g*, 15 min, 4 °C) and the pellet was re-suspended in ice-cold sucrose buffer (20 mM Tris/HCl, 25% (w/v) sucrose, 5 mM ethylenediaminetetraacetic acid (EDTA) at pH 8.0). After 15 min on ice, the solution was centrifuged (8000 × *g*, 15 min, 4 °C) and the pellet was re-suspended in periplasmic lysis buffer (5 mM MgCl_2_, 150 μg/mL lysozyme and 150 μg/mL DNase). The solution was kept cold for 30 min, centrifuged (8000 × *g*, 20 min, 4 °C) and the supernatant was used for further purification according to the protocol described below.

For the biochemical analysis, we produced full-length GbpA, the GbpA LPMO domain, and GbpA variants in minimal media (Sørensen et al., 2023a). Briefly, a 2 mL preculture in LB medium was grown at 37 °C for 6 hours in the presence of 50 μg/mL of kanamycin sulfate in a 17 × 100 mm culture tube at 220 rpm. Then, 200 μL of the preculture were used to inoculate 25 mL of M9glyc+ medium with 50 μg/mL of kanamycin sulfate in a baffled Erlenmeyer flask. A total volume of 1 L of M9glyc+ media contained 19 g K_2_HPO_4_, 5 g KH_2_PO_4_, 9 g Na_2_HPO_4_, 2.4 g K_2_SO_4_, 5.0 g NH_4_Cl, 18 g glycerol, 10 mM MgCl_2_, 1x MEM vitamins, and 1x trace element solution. 0.1 L of 100x trace element solution contained 0.6 g FeSO_4_·7H_2_O, 0.6 g CaCl_2_·2H_2_O, 0.12 g MnCl_2_·4H_2_O, 0.08 g CoCl_2_·6H_2_O, 0.07 g ZnSO_4_·7H_2_O, 0.03 g CuCl_2_·2H_2_O, 0.02 g H_3_BO_4_, 0.025 g (NH4)_6_Mo_7_O_24_·4H_2_O and 17 mM EDTA. Subsequently, the culture was grown at 37 °C and 120 rpm. After 16 hours, 225 mL of fresh M9glyc+ media with 50 μg/mL of kanamycin sulfate were added to the baffled flask. Approximately 2 hours later, when the OD_600_ was between 2 and 3, protein production was induced by adding IPTG to a concentration of 1 mM, and lowering the temperature to 20 °C. Harvesting was performed after 20 hours by centrifugation at 10,000 × *g* for 30 min at 4 °C.

To separate the periplasmatic fraction from the harvested cells, the cell pellet was resuspended in 5 mL per gram of cell paste of a buffered hypertonic solution (Tris-HCl 20 mM pH 8.0, 25% w/v of d-saccharose and 5 mM EDTA). The solution was incubated under gentle stirring for 30 min at 4 °C and centrifuged at 10,000 × *g* for 30 min at 4 °C. The supernatant was kept for further purification and the pellet was resuspended in a hypotonic solution (Tris-HCl 20 mM pH 8.0, 5 mM MgCl_2_, 1 mM phenylmethylsulfonyl fluoride, and 0.25 mg/mL of lysozyme from chicken egg white (Sigma-Aldrich)). The solution was incubated for another 30 min under gentle stirring at 4 °C and centrifuged at 10,000 × *g* for 30 min at 4 °C. The supernatant was also saved for further purification.

The two supernatant fractions were loaded into a HiTrap Q HP 5 mL anion exchange column equilibrated in 20 mM Tris-HCl pH 8.0, 50 mM NaCl and eluted with a linear gradient of 20 column volumes to 400 mM NaCl. The peaks containing the desired protein were concentrated and loaded into a Superdex 75 Increase 10/300 GL column equilibrated in 20 mM Tris-HCl pH 8.0 for a final purification step.

### Multiple sequence alignments

Sequences for the related orthologs in the *Vibrio* species clade were obtained from OrthoDB (Kuznetsov et al., 2023) (group 52685at662 at *Vibrio* level), while the sequence files for all the annotated members of the AA10 family were obtained from Genbank (Benson et al., 2012) based on the annotations in the CAZy database (Drula et al., 2022). Multiple sequence alignments were performed with Clustal Omega (Madeira et al., 2022) with default parameters and manually inspected in Jalview (Waterhouse et al., 2009).

### Differential scanning fluorimetry

Differential scanning fluorimetry (DSF) experiments were carried out in a Prometheus NT.48 system from NanoTemper (NanoDSF), measuring the change in fluorescence by the protein aromatic residues upon thermal denaturation. The samples were buffer exchanged to 20 mM HEPES pH 7.0 with the corresponding salt present at 50 mM with overnight dialysis using Slide-A-Lyzer dialysis cassettes from ThermoFisher Scientific. The pH of the HEPES buffer was adjusted with NaOH to an equivalent final concentration of 7 mM Na^+^ in 20 mM HEPES. Measurements were done in triplicates for GbpA_FL_WT, GbpA_FL_D70A, and GbpA_FL_D70K, whereas GbpA_LPMO_WT was analyzed in duplicates. The obtained values for each sample type were averaged, with the standard deviation between equivalent measurements being lower than 0.3 °C in all cases.

NanoDSF analysis at varying salt concentrations was carried out under the same buffer conditions, 20 mM HEPES pH 7.0. Copper saturation was performed with a 3:1 molar excess of CuCl_2_, followed by incubation for 30 min at room temperature. The unbound copper was then eliminated through buffer exchange. Measurements were done in triplicates in the conditions for which affinity constants could be calculated. Data analysis, fitting, and plotting were performed using GraphPad Prism. The binding curves were fitted following a single-site ligand-binding model, as described by Vivoli et al. (2014).

### Chitin-binding assay

Chitin-binding assays were performed with copper-saturated GbpA_FL_. The protein was saturated with a 3:1 molar excess of CuCl_2_, incubated for 30 min at room temperature and desalted using alternating protein concentration and dilution steps in Viva-spin 20 centrifugal units (Sartorius). Reactions were run in a final volume of 500 μL in 20 mM HEPES pH 8.0 and a protein concentration of 12 μg/mL. Salts were added to a final concentration of 10 *K*_d_ for both Ca^2+^ (2.2 mM) and Mg^2+^ (23 mM). β-chitin particles (Glentham Life Sciences, GC2112) at a concentration of 10 mg/mL were used as a substrate, milled on a cutter mill (Retsch, Haan, Germany) and subsequently a planetary ball mill (Retsch, Haan, Germany), and sieved to a final size of 75–200 μm. The reactions were carried out at 23 °C with intense shaking (800 rpm) to make sure that the chitin suspension would stay homogenous. At given timepoints, aliquots of 50 μL were filtered using a 96-well filter plate (Merck Millipore) to separate the unbound protein from the insoluble chitin. The filtered protein sample was diluted to a final volume of 100 μL in a 96-well plate, and A595 and A450 were recorded on a Varioskan Lux plate reader (ThermoFisher Scientific). Protein concentration was measured using a modified version of the linearized Bradford protein assay (Ernst and Zor, 2010).

### Chitin degradation activity assays

For activity assays, the different GbpA variants were copper-saturated with a 3:1 molar excess of CuCl_2_ and incubated for 30 min at room temperature, with subsequent desalting. Reactions were carried out in triplicates in a total volume of 500 μL in 20 mM HEPES pH 8.0, with a final enzyme concentration of 0.5 μM. Salts were added to the reaction at the desired concentration. β-chitin particles (Glentham Life Sciences, GC2112) at a concentration of 10 mg/mL were used as a substrate, milled on a cutter mill (Retsch, Haan, Germany) and subsequently a planetary ball mill (Retsch, Haan, Germany), and sieved to a final size of 75–200 μm. The reactions were carried out at 37 °C and 800 rpm in 2 mL Eppendorf tubes in a Thermomixer (Eppendorf). Before starting the reaction, the protein was allowed to get to a binding equilibrium with chitin for 20 min. For the reactions run under monooxygenase conditions (without added H_2_O_2_), 1 mM ascorbic acid was used to start the LPMO reaction. For the reactions run under peroxygenase conditions, ascorbic acid was added to a final concentration of 50 μM (simultaneously with H_2_O_2_). At the required time points, 50 μL aliquots were filtered on a 96-well filter plate (Merck Millipore) to remove the insoluble substrate and stop the reaction. Thereafter, 25 μL of the filtrate were incubated overnight with 1 μL of chitobiase added to a final concentration of 0.27 μM in order to convert the solubilized oxidized oligosaccharides to chitobionic acid. LPMO activity was assumed based on quantification of the oxidized monosaccharide based on standard curves for chitobionic acid using a Rezex RFQ-Fast acid H^+^ (8%) 7.8 × 100 mm column (Phenomenex, Torrance, CA) at 85 °C on a Dionex Ultimate 3000 UPLC (Dionex, Sunnyvale, CA, USA) using H_2_SO_4_ 5 mM as a mobile phase at a flow rate of 1 mL/min (Mekasha et al., 2020).

### Small-angle X-ray scattering

SAXS data were collected at the BM29 BioSAXS beamline (Pernot et al., 2013) at the European Synchrotron Radiation Facility (ESRF) in Grenoble, France. A wavelength λ of 0.992 Å was used, and scattering intensities I(q) were recorded as a function of the scattering vector q = (4π/λ) sin(θ), where 2θ is the scattering angle. The acquisition was done in the q-range 0.0037–0.5 Å^-1^. 10 frames were collected per sample and 20 for each buffer. Before further processing, frames with radiation damage were discarded.

The DOI associated with the data collection is 10.15151/ESRF-ES-649173298. Samples were measured in 50 mM Bis-Tris propane pH 7.0 and 500 mM of the respective salts at 37 °C. Data with 10 mM NaCl were also recorded for comparison. However, as GbpA was particularly susceptible to radiation damage in the presence of CaCl_2_, parameters were optimized to limit this issue, necessitating use of HEPES buffer at 20 mM and pH 7.0, in which CaCl_2_ is present at only 50 mM. Additionally, we attenuated the beam; statistics for the CaCl_2_ data are hence weaker. The scattering contribution of the buffer was subtracted from measurements, and the data were calibrated to absolute scale using the scattering of H_2_O as a standard. Data were collected for GbpA at different concentrations (0.125, 0.25, 0.5, 1, 2, and 4 mg/mL), and the datasets merged with *ALMERGE* (Franke et al., 2012) from the *ATSAS* package (Manalastas-Cantos et al., 2021), generating datasets extrapolated to zero solute concentration. *R_g_* values were obtained through Guinier analysis using home-written MATLAB scripts. I(q) versus q plots and dimensionless Kratky plots were generated with MATLAB.

### Protein crystallization and X-ray data collection and refinement

Before crystallization of the LPMO domain, the protein was treated with a 3:1 molar excess of CuCl_2_ and incubated for 30 min at room temperature, then desalted and concentrated to 20 mg/mL. Crystals of good diffraction quality were obtained from 0.2 M calcium chloride dihydrate, 0.1 M HEPES pH 7.0, 20% w/v PEG 6000 or 0.1 M potassium thiocyanate, 30% PEG 2000 monomethyl ether, respectively. Diffraction data to 1.8 Å resolution were collected at beam line BM30 at the ESRF (Grenoble, France) from the crystals obtained from the calcium-containing conditions. X-ray data were auto-processed at the ESRF by the *EDNA* pipeline (Incardona et al., 2009). The structure was phased by molecular replacement (MR) with the *PHENIX* crystallographic software package (Liebschner et al., 2019), using domain 1 of the GbpA structure (Protein Data Bank (PDB) ID:2XWX; D1-3) (Wong et al., 2012) as a search model, and refined in alternating cycles of manual model building and refinement with *PHENIX* (Liebschner et al., 2019) and *Coot* (Emsley et al., 2010). Water molecules were added at later stages of the refinement, initially using the automated water-picking routine of *PHENIX* (Liebschner et al., 2019). These sites were then inspected individually. Strong spherical electron density was found near the active site. While lighter ions or molecules (e.g., sodium or water) could not explain the strong electron density, leaving a residual difference density peak in the binding site after refinement, calcium accounted for the electron density very well. Moreover, the coordination geometry (pentagonal bipyramid) and bond distances to neighboring atoms of the residues and water molecules further strengthened the interpretation as a calcium site (Table S1).

For crystals obtained from the potassium-containing condition, diffraction data to 1.5 Å resolution were collected at ESRF beamline BM14. The structure was phased with MR, and modeled and refined similarly to the calcium-bound structure. To aid structural analysis, in particular of the cation-binding site, we collected a complete data set with supporting anomalous signal. The anomalous cross-correlation (CC) coefficient showed significant anomalous data to 3.9 Å (anomalous CC of 11 for the resolution shell 4.7–3.9). Anomalous map coefficients were calculated using phenix.maps (Liebschner et al., 2019) with default settings utilizing the full resolution range of the dataset. Strong density was found in the same site previously occupied by calcium, in this structure with octahedral geometry. The identity of potassium over other ions or molecules, such as sodium or water, was confirmed by the presence of a strong anomalous signal (Figure S1). Furthermore, the bond distances to atoms in the coordination sphere supported this identity (Table S1).

The cations in both structures were refined with full occupancies. X-ray data collection, processing, and refinement statistics are summarized in Table 1.

**Table 1.** X-ray data collection and refinement statistics

(a) Data collection		
	GbpA_LPMO_ (Ca^2+^)	GbpA_LPMO_ (K^+^)
Beamline	BM30A	BM14
Wavelength (Å)	0.9797	0.9537
Resolution range^a^	41.0–1.8 (1.90–1.80)	46.6–1.5 (1.59–1.50)
Space group	*P* 2_1_	*P* 4
Cell parameters		
a, b, c (Å)	40.6, 45.2, 99.6	46.6, 46.6, 157.4
α, β, γ (°)	90, 101.1, 90	90, 90, 90
*R*_meas_ (%)	0.197 (0.969)	0.062 (0.263)
CC_1/2_	0.993 (0.703)	0.999 (0.973)
Mean I/σ(I)	7.8 (1.8)	28.4 (7.9)
Completeness (%)	99.5 (98.5)	98.5 (90.8)
Multiplicity	4.1 (4.1)	13.4 (10.9)
Unique reflections	33,236 (5267)	52,428 (7798)

(b) Refinement		
	GbpA_LPMO_ (Ca^2+^)	GbpA_LPMO_ (K^+^)
Resolution (Å)	34.3–1.80	40.1–1.50
*R*_work_/ *R*_free_	0.179/0.228	0.164/0.192
Macromolecules/asymmetric unit	2	2
Number of non-hydrogen atoms		
Protein	2798	2828
Ligands	4	24
Waters	512	565
*B*-factors (Å^2^)		
Protein	14.3	12.0
Ligands	12.4	17.9
Waters	27.0	24.5
r.m.s.d. from ideal values		
Bond length (Å)	0.005	0.007
Bond angles (deg)	0.72	0.81
Ramachandran plot		
Favored (%)	96.9	97.8
Outliers (%)	3.1	2.2
PDB ID	7PB7	7PB6

a Data in parentheses refer to high-resolution shell.

## Results

### New cation-binding site identified close to LPMO active site

**Figure 2.**
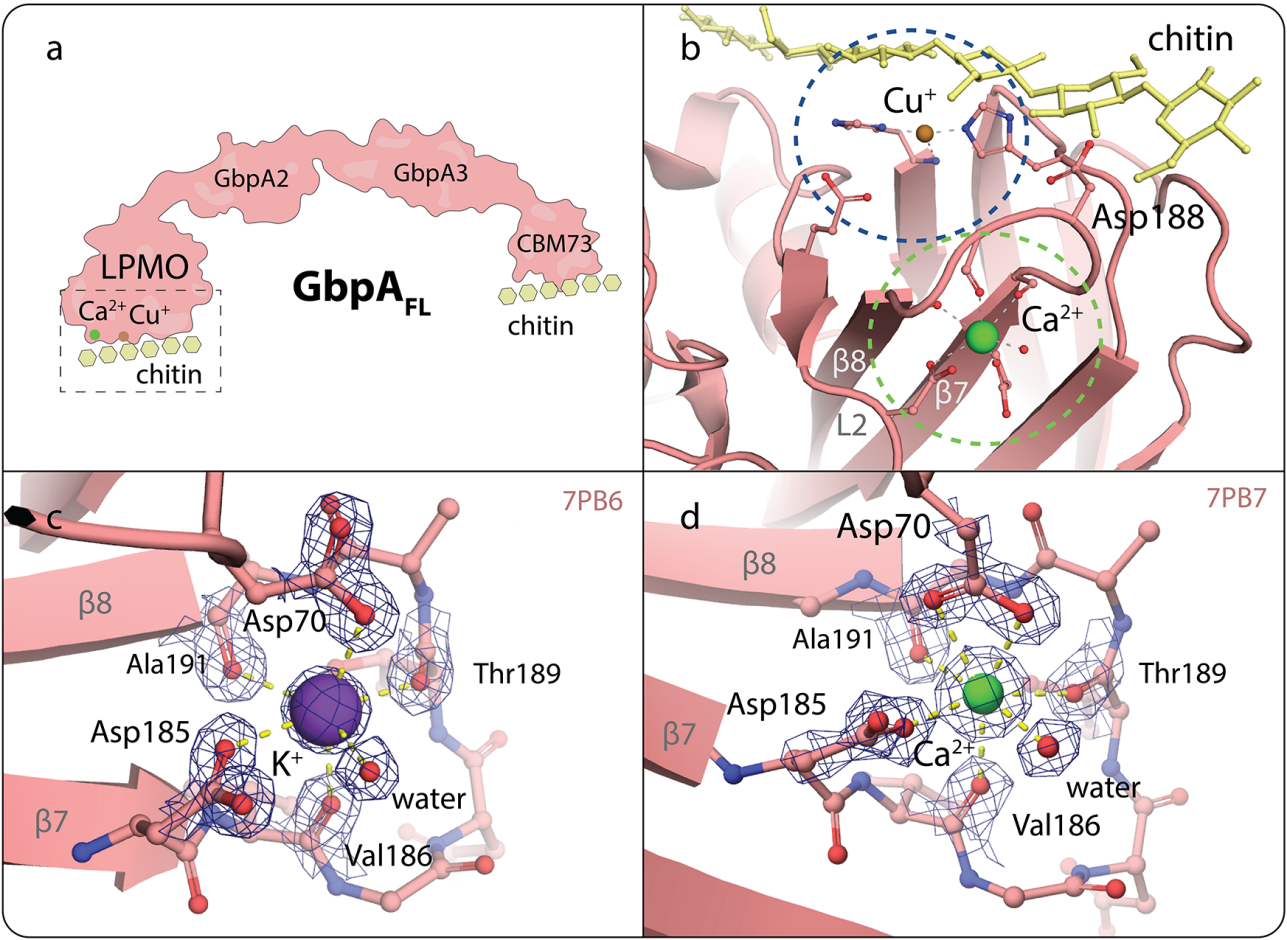
**LPMO cation-binding sites. a**. Schematic representation of GbpA and its domains. The dashed box indicates the region shown in panel B (in different orientations). **b**. Overview of the newly identified cation-binding site (green dashed circle) in proximity of the GbpA active site (blue dashed circle) and carbohydrate-binding surface. Ca^2+^ is represented as green sphere; the active-site copper ion as bronze sphere, bound to the histidine-brace motif characteristic of LPMOs. The carbohydrate substrate (yellow) has been manually modeled in its expected position based on (Tandrup et al., 2020), taking into account information from (Bissaro et al., 2018). Important residues belonging to the active site, the chitin-binding surface (in particular Asp188), and the newly identified metal-binding site are depicted in stick representation. The L2 loop and β-strands 7 and 8 are labeled **c.** Close-up view of the cation-binding site featuring K^+^ (purple sphere with tetragonal bipyramidal coordination indicated by dashed lines), with σ_A_-weighted 2m*F*o-D*F*c map (blue mesh) contoured at 2σ (PDB ID: 7PB6; this work). **d.** Close-up view of the cation-binding site featuring Ca^2+^ (green sphere with pentagonal bipyramidal coordination), with σ_A_-weighted 2m*F*o-D*F*c map contoured at 2σ (PDB ID: 7PB7; this work). Note the different side chain conformations of Asp70, which results in different metal ion coordination of K^+^ compared to Ca^2+^ (monovalent instead of divalent interaction).

The LPMO domain of GbpA (GbpA_LPMO_) was saturated with copper, and single crystals suitable for diffraction were obtained in the presence of calcium or potassium ions. The crystal structures of the LPMO-calcium and LPMO-potassium complexes were refined to 1.8 Å and 1.5 Å, respectively, with *R*/*R*_free_ values of 0.179/0.228 and 0.164/0.192 (Table 1). The structures are deposited in the PDB (Berman et al., 2000) with accession IDs 7PB7 (Ca^2+^) and 7PB6 (K^+^). In both structures, a cation-binding site was identified close to the copper-binding active site of the LPMO (Figure 2a and 2b). The nature of the cation was predicted to be the dominant cation species present in the experiments (0.2 M calcium and 0.1 M potassium, respectively), and further validated based on geometry analysis, binding distances, electron density peak height, and anomalous diffraction analysis (see *Methods* for details). The cation-binding site is formed mainly by the loop located between strands 7 and 8 of the core β-sandwich characteristic of LPMOs (Vaaje-Kolstad et al., 2017). This includes the backbone carbonyl groups of Val186, Thr189, and Ala191 as well as the side chain carboxylate of Asp185. In addition, coordination by the side chain carboxylate of Asp70, located in the L2 region (Vaaje-Kolstad et al., 2017), is key to binding. The coordination geometry for K^+^ is that of a tetragonal bipyramid, that is, octahedral (Figure 2c), whereas Ca^2+^ coordination is characterized by a distorted pentagonal bipyramid, with the two carboxyl oxygens of Asp70 involved in the interaction (Figure 2d). The distances (Table S1) as well as the coordination geometries are consistent with the expected values for the two metal ions (Harding, 1999, 2001; Zheng et al., 2008). In particular, pentagonal bipyramidal coordination is typical for divalent calcium and unusual for many other ions (McPhalen et al., 1991; Strynadka and James, 1991). A water molecule completes the coordination sphere of both cations.

Since the presence of a cation fixes the loop formed by residues 186–189 to the sequentially distal Asp70 located in the L2 region (Vaaje-Kolstad et al., 2017) (Figure 2c and 2d), we hypothesized that it could confer stabilization of the LPMO domain. Furthermore, the same loop region is part of the LPMO carbohydrate-binding surface, with Asp188 known to be involved in chitin binding (Figure 2b) (Bissaro et al., 2018).

### Effect of metal ions on GbpA thermostability

**Figure 3.**
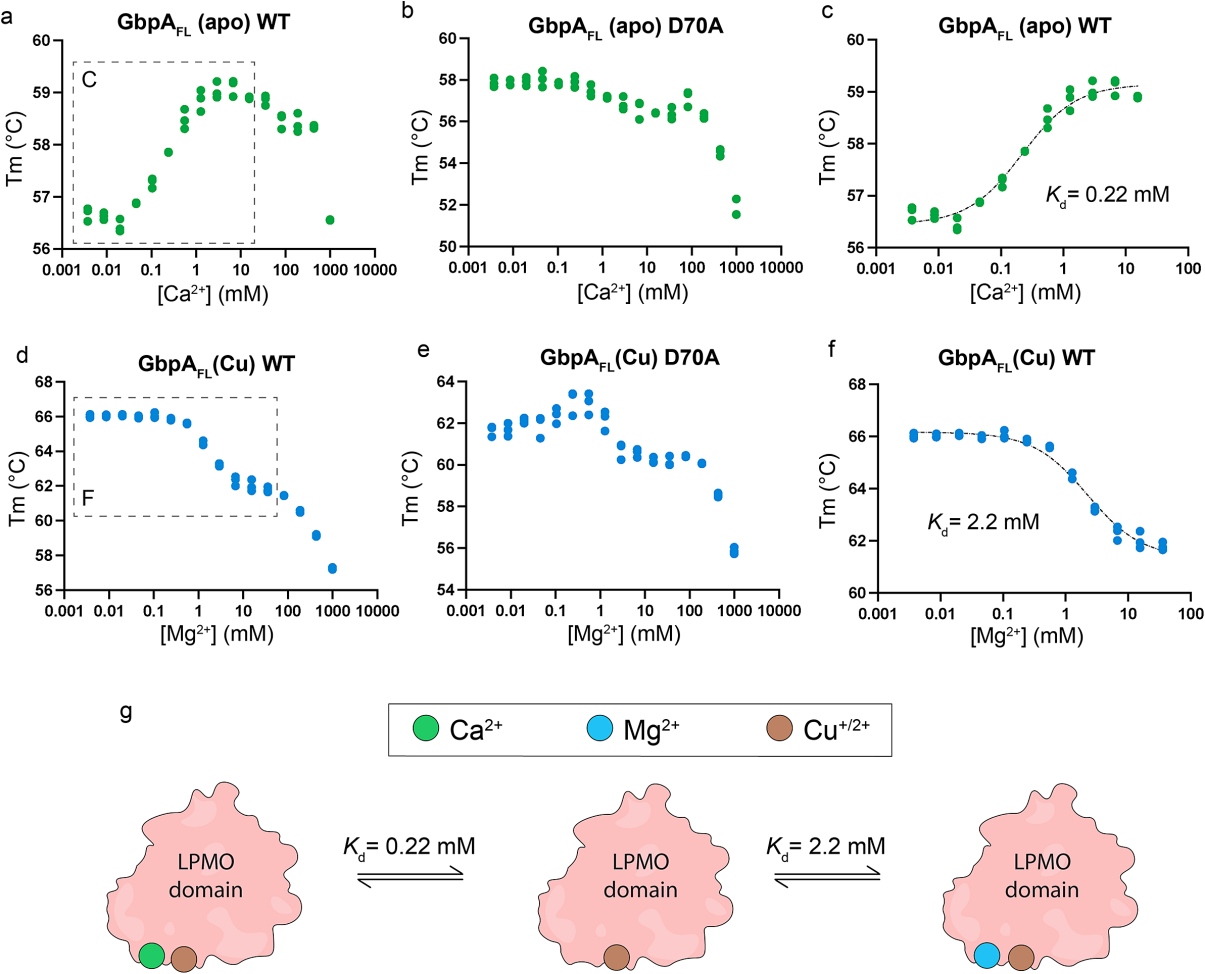
**Specific effects of divalent ions on GbpA stability**. **a–c**. Effects of calcium ion concentration on apo GbpA_FL_ stability. Note the specific and saturable stabilization at physiological ion concentrations observed only for wild-type (WT) GbpA. **d–f.** Effects of magnesium ion concentration on copper-saturated GbpA_FL_. Specific and saturable destabilization is observed for WT GbpA. **g**. Graphical representation of GbpA metal-binding states. Each state potentially has different substrate-binding affinities and catalytic activities. For clarity, only the representation of the GbpA LPMO domain is shown, excluding the second, third and fourth domains. The dissociation constant of copper-saturated GbpA for calcium is assumed to be the same as for the apo protein.

DSF was used to study the effect of different cations on the GbpA LPMO domain (GbpA_LPMO_) and on the full-length protein (GbpA_FL_). A significant stabilizing effect was observed in the presence of calcium, with an increase in melting temperatures (ΔT_m_) of 3.4 °C and 3.8 °C for GbpA_LPMO_ and GbpA_FL_, respectively (Table S2). The analysis was extended to different calcium concentrations (Figure 3) for both apo and copper-saturated GbpA_FL_. Here, only apo GbpA showed saturable binding to calcium with a calculated dissociation constant (*K*_d_) of 0.22 mM (Figure 3a and 3c). To probe if stabilization by calcium is attributed to the identified binding site, Asp70 was substituted with Ala in GbpA_FL_. GbpA_FL_D70A exhibited similar thermal stability as wild-type (WT) GbpA in the absence of calcium, but completely lost the additional stabilizing effect caused by calcium (Figure 3b, Table S2).

When analyzing magnesium binding to GbpA at different ion concentrations, a saturable destabilizing effect was observed for the protein that contained copper in the active site (*K*_d_ = 2.2 mM; Figure 3d and 3f). Magnesium most likely binds to the same site as calcium, since this destabilizing effect is not clearly observed for the GbpA_FL_D70A variant (Figure 3e).

At high concentrations, divalent cations appear to cause a general nonspecific destabilizing effect, as observed for all magnesium and calcium conditions (Figure 3, Figure S2). In contrast, monovalent cations appear to confer only small stabilizing or destabilizing effects on GbpA, with equivalent trends observed for Na^+^ and K^+^ (Table S2, Figure S2). The changes in stability induced by monovalent and divalent cations do not affect the overall shape of GbpA, as shown by SAXS (Figure S3). The shape of GbpA was similar for all the tested salts (NaCl, KCl, CaCl_2_, and MgCl_2_), with a radius of gyration (*R_g_*) of approximately 38–39 Å.

### The identified cation-binding site mimics conserved contacts in related LPMOs

**Figure 4.**
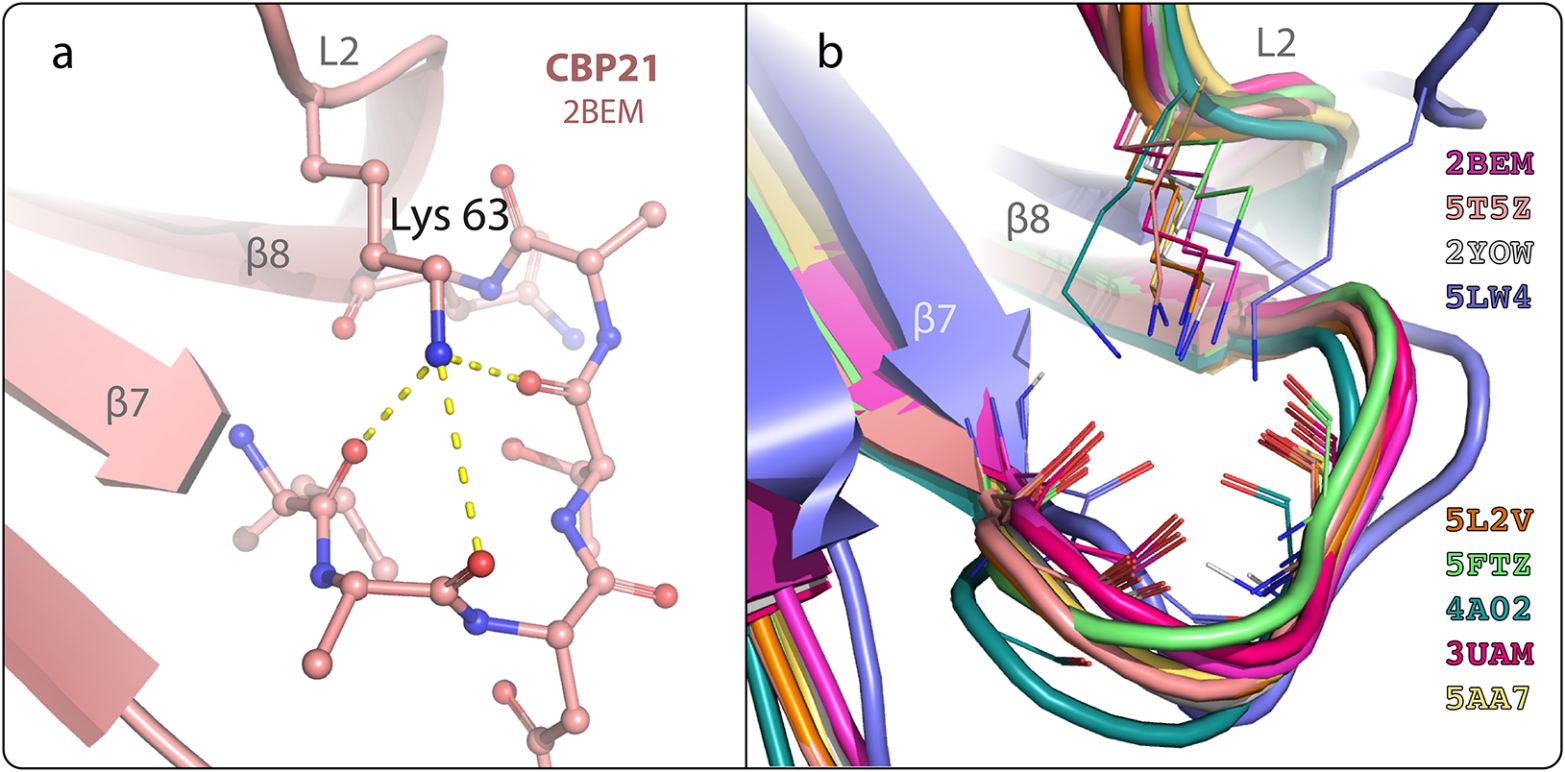
**Conserved contacts between the L2 loop lysine and the β7-β8 loop**. **a**. *Serratia marcescens* chitin-binding protein 21 (CBP21; PDB ID: 2BEM (Vaaje-Kolstad et al., 2005)), with detailed structural interactions of L2 loop residue Lys63 that stabilizes the loop between β-strands 7 and 8 depicted in Figure 2. **b.** Structural alignment of AA10 LPMOs. The alignment includes all AA10 LPMO structures currently accessible in the PDB that exhibit a lysine residue in the L2 loop. The structures are color-coded, with PDB IDs given to the left.

The cation-binding site in GbpA is formed mostly by backbone interactions, except for Asp70 in the L2 loop and Asp185 in the loop between β-strands 7 and 8 (Figure 2). Asp70 is highly conserved among GbpA of *V. cholerae* strains (Stauder et al., 2012), even though this is not the case for GbpA orthologs among the *Vibrio* genus in general (sequence alignment not shown). Extending the multiple sequence alignment to more phylogenetically distant LPMOs (classified as AA10 in the CAZy database), we noticed that in 45% of the annotated sequences, the residue found in the position equivalent to Asp70 of GbpA is a lysine. Lysine is also the most common residue in that position for the AA10 LPMO structures deposited in the PDB (Figure S4). Interestingly, the positively charged lysine ammonium group occupies the position of the metal ions and interacts with the backbone residues that constitute the metal-binding site. This is exemplified for chitin-binding protein 21 (CBP21) from *Serratia marcescens* in Figure 4a, chosen as a representative example of AA10 LPMO structures featuring a lysine in the L2 region loop. A structural alignment of all the experimentally obtained AA10 LPMO structures with lysine in the position equivalent to Asp70 in GbpA is shown in Figure 4b.

In order to better understand the effect that the emergence of the binding site might have had for GbpA, we engineered an additional GbpA_FL_ variant, D70K. As expected, this substitution eliminated the specific stabilizing effect by calcium (Table S2). However, we did not observe any significant compensating effect in the form of general protein stabilization in the absence of salts (ΔT_m_ = 0.4 °C, shown in Table S2). Therefore, the presence of the positively charged ammonium group in the metal-binding site did not mimic the binding of calcium but is more similar to monovalent ions like potassium and sodium, which have little effect on GbpA stability (Table S2, Figure S2).

### Ca^2+^ and Mg^2+^ increase the catalytic activity of GbpA mostly through nonspecific mechanisms

**Figure 5.**
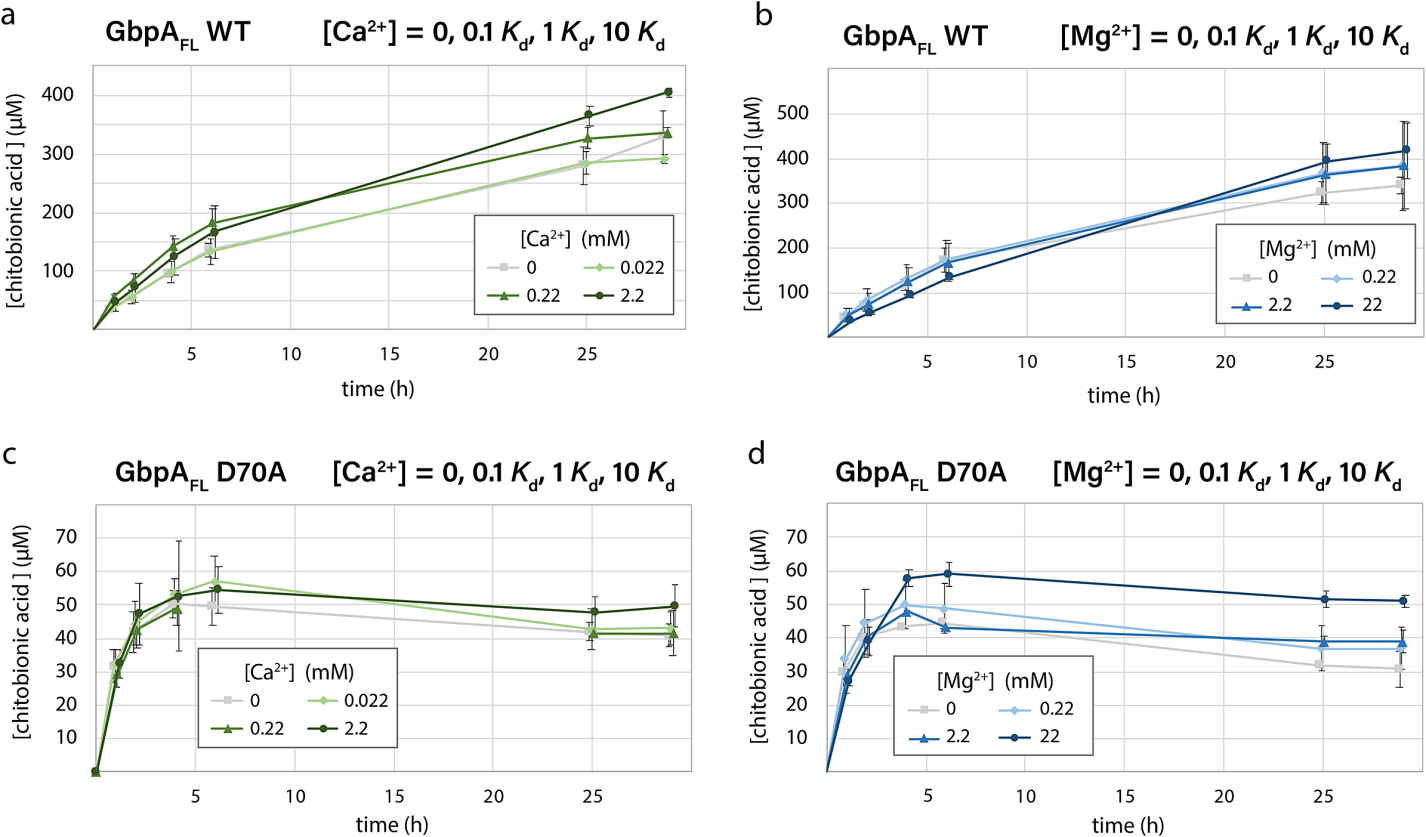
**Catalytic activity of GbpA in the presence of salts**. GbpA_FL_ (WT and D70A) were probed for production of chitobionic acid in the presence of different concentrations of calcium and magnesium. Ascorbic acid was used for LPMO reduction and H_2_O_2_ was generated by autooxidation of ascorbic acid. For Ca^2+^; 0.1 *K*_d_ = 0.022 mM, 1 *K*_d_ = 0.22 mM, and 10 *K*_d_ = 2.2 mM. For Mg^2+^; 0.1 *K*_d_ = 0.22 mM, 1 *K*_d_ = 2.2 mM, and 10 *K*_d_ = 22 mM. Experiments were performed in triplicates and the error bars refer to standard deviations.

Next, we studied the effect of magnesium and calcium ions on GbpA-catalyzed chitin oxidation *in vitro* (Figure 5a and 5b). The catalytic activity was measured in the presence and absence of Ca^2+^ and Mg^2+^. The salt concentrations applied were 0.1, 1, and 10 x *K*_d_. The production of soluble oxidized chitooligosaccharides was studied under conditions where the H_2_O_2_ co-substrate was provided either directly or indirectly (Bissaro et al., 2017). When H_2_O_2_ was provided indirectly by ascorbic acid autooxidation, marginally more oxidized products were produced both for GbpA_FL_ and the GbpA_FL_ D70A variant in the presence of calcium and magnesium than in the absence of divalent ions (Figure 5). However, since calcium/magnesium binding is abolished in the GbpA_FL_ D70A variant (Figure 3), the increase in activity appears to be mainly due to unspecific effects. Regardless, the catalytic activity of the engineered GbpA_FL_ variants (D70A and D70K) was much lower than for the WT protein (Figure 5c and 5d), limiting their use as a negative control. The decrease in activity observed for these variants (after 3 hours) indicates lower protein stability, possibly related to oxidative inactivation, a phenomenon that is common for LPMOs (Bissaro et al., 2017; Loose et al., 2018; Stepnov et al., 2022).

Catalytic activity was also measured for the construct GbpA_LPMO_, which lacks the fourth domain (as well as GbpA domains 2 and 3). The fourth domain is a family 73 carbohydrate-binding module (CBM73) that is important for chitin binding (Wong et al., 2012). In the absence of domain 4 (GbpA_LPMO_), the catalytic activity is almost fourfold lower than for WT GbpA (Figure S5). This suggests that chitin binding is important for GbpA activity, most likely by increasing the concentration of the enzyme on the insoluble substrate and through protection of the enzyme from oxidative damage, as shown for other LPMOs (Forsberg and Courtade, 2023).

When directly adding H_2_O_2_ as a co-substrate, LPMO activity is often limited by inactivation through oxidative damage (Petrović et al., 2018). Therefore, we analyzed if the presence of ions in the studied metal-binding site could have a protective effect against oxidative damage. We then replicated the experiments performed by Petrović et al. (2018), where the methylation of a histidine side chain in the active site of another LPMO was found to provide protection against oxidative damage. In our case, however, the presence of calcium did not increase the tolerance to H_2_O_2_. In other words, at a given concentration of H_2_O_2_, the addition of calcium did not result in increased production of oxidized soluble saccharides (Figure S6).

## Discussion

Here, we report the discovery of a metal-binding site in GbpA close to the LPMO active site. We determined the crystal structures of the GbpA LPMO domain in complex with either calcium or potassium ions to high resolution and tested the effect of different metal ions on GbpA thermostability. Monovalent cations such as Na^+^ and K^+^ were found to nonspecifically stabilize GbpA, whereas Ca^2+^ and Mg^2+^ were found to have specific effects on GbpA stability – with calcium stabilizing and magnesium destabilizing the protein. Stabilization by calcium (in the high-micromolar to low-millimolar range) was only observed for GbpA in the apo state, and not for the copper-saturated protein. However, the crystal structure determined by us (PDB ID: 7PB7) clearly shows that calcium can bind to GbpA also in the presence of copper. We therefore assume that the stabilizing effect of calcium is likely shielded by the strongly stabilizing copper ion, which is reported to bind to the histidine brace with high affinity, in the low-nanomolar range (Ipsen et al., 2021). In contrast, magnesium was found to destabilize GbpA in the low-millimolar range. The origin of the opposite stabilization effects by the two divalent ions remains unknown. The two ions are likely coordinated differently (as observed for K^+^ compared to Ca^2+^), with potential changes in structure and dynamics. Destabilization by Mg^2+^ binding could imply an increase in flexibility that may be required for improved functionality.

Whereas the presence of Ca^2+^ and Mg^2+^ ions affected GbpA stability, the cations did not seem to specifically influence the protein’s catalytic properties, that is, the rates of chitin oxidation or the total amount of reaction products (at least not under the conditions tested). Nevertheless, the disruption of the metal-binding site (D70→A) in full-length GbpA resulted in the rapid loss of catalytic activity (Figure 5). This suggests that the structural features stabilized by Ca^2+^ binding are important either for active site integrity, for binding to chitin or both. The similar initial catalytic rates of GbpA WT and D70A variant, and abrupt activity loss of the latter, can indicate a decrease in oxidative stability, possibly due to destabilization of the histidine brace. Such a scenario would cause an increase in free copper that can accelerate H_2_O_2_ generation, as shown by others (Stepnov et al., 2022). Indeed, we show that GpbA is very sensitive to free H_2_O_2_ (Figure S6). Furthermore, the significant activity loss of the variants also limits their suitability as a negative control to study the specificity of the effects of calcium and magnesium on the catalytic activity of GbpA. It is thus fully possible, and even likely, that the cations have important regulatory roles through the newly discovered binding site, even though our experiments did not allow us to distinguish between specific and unspecific effects on protein activity.

How could such regulation occur? GbpA is known to bind chitin through its LPMO domain 1 and CBM73 domain 4 (Wong et al., 2012), whereas binding to mucins appears to only involve the LPMO domain (Wong et al., 2012); however, relatively little detail is known about these interactions at the molecular level (Sørensen et al., 2023b). An additional challenge is assessing what ionic compositions *V. cholerae* might be exposed to in its local microenvironment during environmental survival and intestinal colonization is a complex task.

Calcium and magnesium concentrations in aquatic environments are generally low, and limited by the solubility of their carbonate salts (Bentov et al., 2016) to around 0.13 mM for calcium and 1.4 mM for magnesium ions (both measured at 25 °C). Interestingly, the dissociation constants obtained for both salts are approximately twice their solubility in the ocean, with *K*_d_ = 0.22 mM and 2.2 mM for calcium and magnesium ions, respectively. Although dissociation constants calculated by thermal shift analysis are obtained at the nonphysiological melting temperature of the protein and could thus differ significantly from the effective values, it seems that GbpA would require to be exposed to higher ion concentrations than those generally present in the marine environment. However, the chitinous exoskeleton of crustaceans is mineralized mostly with calcium and – in smaller amounts – magnesium amorphous precipitates (Raabe et al., 2006). Therefore, GbpA is most likely exposed to significantly higher local ion concentrations when binding to chitin of crustaceans. Furthermore, the amount of mineralization and the nanostructure of the ionic precipitate varies widely for different body parts (Fabritius et al., 2011) as well as among different species of crustaceans. It is thus plausible that GbpA could establish preferential binding to certain surfaces or organisms, paving the way toward colony and biofilm formation. Chitin binding increases chitin oxidation and degradation by GbpA, supplying the microcolony with nutrients; and the interplay between calcium and magnesium may facilitate binding and release during chitin processing. Calcium has already been shown to negative influence in biofilm formation in *V. cholerae* by affecting gene expression (Bilecen and Yildiz, 2009). Furthermore, calcium-binding domains have been described for the *V. cholerae* biofilm matrix proteins RbmC and Bap1 (Fong and Yildiz, 2007), pointing to complex and still unknown regulation mechanisms.

Conversely, in the mammalian gut, where GbpA is involved in pathogenesis, total calcium and magnesium levels are likely to be lower than in chitin. The concentrations of these cations in the human serum are 2.2–2.6 mM for calcium and 0.7–1.1 mM for magnesium (Jahnen-Dechent and Ketteler, 2012); however, this does not translate directly to the concentrations in the gut. Ion concentrations are difficult to estimate due to the highly complex environment and composition of the organ, where local ion concentrations may vary and depend on factors such as diet and absorption by the host (Hoenderop et al., 2005), as well as interactions with the native microbiota. Nevertheless, calcium concentrations in the gut are sufficiently high to be relevant for the conformation and mechanical properties of mucins (Ambort et al., 2012; Hughes et al., 2019). Mucins contain multiple calcium-binding sites in their von Willebrand factor type D and CysD domains and expand in the absence of these ions (Javitt et al., 2020). We hypothesize that calcium binding to GbpA could represent a competition mechanism by which the presence of GbpA in the gut could affect mucin conformations, aiding colonization by *V. cholerae* through a less dense mucin structure and possibly facilitating toxin entry.

The role of K^+^ remains enigmatic. In terms of protein stability, the presence of a potassium ion in the metal-binding site might be equivalent to that of the lysine residue from loop L2, which occupies the metal-binding site in some AA10 LPMOs. However, the relatively conserved nature of the lysine could indicate an important role in other AA10 LPMOs. We hypothesize that monovalent ions, generally more soluble and abundant (Wang et al., 2020), could represent opportunistic binders in the absence of calcium or magnesium and thus participate in a regulation mechanism by ion exchange when calcium or magnesium concentrations are below a certain threshold.

Although the cation-binding site is rare among orthologs of GbpA from the *Vibrio* genus, it is conserved among all *V. cholerae* strains analyzed by Stauder et al. (2012), both from environmental and pathogenic origins. This suggests that the binding site is likely an important adaptation to the lifestyle of these bacteria, whereas other species, featuring a conserved lysine at this position, lack such a regulation mechanism. This supports the hypothesis that the local structure and organization of the region involving the L2 loop as well as the loop connecting strands 7 and 8 are important for these LPMOs, with potential implications for binding and catalysis.

Summarizing, we report the discovery of a second cation-binding site in GbpA, which is in close proximity of the active and substrate-binding sites. We show that calcium and magnesium specifically affect GbpA stability, suggesting that ion binding may modulate binding and catalytic activity *in vivo*, with potentially important consequences for *V. cholerae* infectivity and survival in different environmental niches.

## Supporting information

Montserrat-Canals et al. supplementary material Montserrat-Canals et al. supplementary material

## Data Availability

Atomic coordinates and structure factors were deposited in the PDB with accession codes 7PB6 (potassium-GbpA_LPMO_) and 7PB7 (calcium-GbpA_LPMO_).

## Supplementary material

The supplementary material for this article can be found at http://doi.org/10.1017/qrd.2024.14.
