# Supplementary material for: Calcium-binding site in AA10 LPMO from Vibrio cholerae suggests modulating effects during environmental survival and infection
Source: QRB Discov. 2024 Dec 26;5:e12. doi: 10.1017/qrd.2024.14 (PMC11729483; doi:10.1017/qrd.2024.14)
Supplement: Montserrat-Canals et al. supplementary material [file S2633289224000140sup001.pdf]

SUPPLEMENTARY INFORMATION

Calcium binding site in AA10 LPMO from *Vibrio cholerae* suggests modulating effects during environmental survival and infection

Mateu Montserrat-Canals[1,2#], Kaare Bjerregaard-Andersen[2§#], Henrik Vinther Sørensen[2], Eirik Kommedal[3], Gabriele Cordara[2], Gustav Vaaje-Kolstad[3], Ute Krengel[2]*

[#] These authors have contributed equally

[1] Centre for Molecular Medicine Norway, University of Oslo, NO-0318 Oslo, Norway

[2] Department of Chemistry, University of Oslo, NO-0315 Oslo, Norway

[3] Faculty of Chemistry, Biotechnology and Food Science, Norwegian University of Life Sciences (NMBU), NO-1433 Ås, Norway

[§] Present address: Ottilia vej 9, H. Lundbeck A/S, 2500 Valby, Denmark

[*]Correspondence: Ute Krengel (ute.krengel@kjemi.uio.no; +47-22855461)

Contents:

Table S1. Donor-metal distances in the newly discovered cation-binding site

Table S2. Thermostability measurements by differential scanning fluorimetry

Table S3. Fitting of dissociation constants (K_d)

Figure S1. Anomalous electron density of K^+

Figure S2. Effect of different cations on GbpA stability

Figure S3. SAXS data for GbpA in the presence of different cations

Figure S4. Multiple sequence alignment of AA10 LPMOs

Figure S5. Effect of salts on GbpA catalytic activity without externally added H_2O_2

Figure S6. Effect of salts on GbpA catalytic activity with H_2O_2 added as co-substrate, and binding to chitin

References

Table S1. **Donor-metal distances in the newly discovered cation-binding site**

	Donor	Ca^{2+} Measured (Å) PDB ID: 7PB7 (Ca^{2+})	Ca^{2+} Expected (Å) (Ca^{2+})	K^{+} Measured (Å) PDB ID: 7PB6 (K^{+})	K^{+} Expected (Å) (K^{+})
**Asp70**	carboxylate oxygen 1	2.46	2.4-2.7	2.82	2.6-2.9
	carboxylate oxygen 2	2.50	2.4-2.7	–	–
**Asp185**	carboxylate oxygen 1	2.30	2.3-2.5	2.71	2.6-2.9
	carboxylate oxygen 2	4.10	3.0-4.6	–	–
**Val186**	backbone carbonyl	2.39	2.3-2.5	2.55	2.6-2.9
**Thr189**	backbone carbonyl	2.41	2.3-2.5	2.76	2.6-2.9
**Ala191**	backbone carbonyl	2.58	2.3-2.5	2.79	2.6-2.9
**Water**	H_2O	2.41	2.3-2.5	2.77	2.5-2.9

Measured and expected distances for the metal ions observed in the newly described cation-binding site. For calcium, expected values were obtained from surveys by Harding (1999 and 2001), and for potassium, from a compilation by Zheng *et al.* (2008) (corresponding to the PDB medium-resolution dataset). All the carboxylate donors are monodentate except for Asp70 coordinating calcium. The only outlier from the expected distance range is the backbone carbonyl of Ala191 coordinating calcium. The increased distance is probably a result of the restrictions imposed by the first β-strand, which starts with Ala191.

Table S2. **Thermostability measurements by differential scanning fluorimetry**

	**$GbpA_{FL}$ WT**	**$GbpA_{LPMO}$ WT**[b]	**$GbpA_{FL}$ D70A**	**$GbpA_{FL}$ D70K**
**No salt**[a]	54.4 ± 0.01 °C	56.4 °C ± 0.04 °C	56.8 °C ± 0.09 °C	54.8 ± 0.24 °C
**$CaCl_2$**	3.8 °C	3.4 °C	-2.0 °C	-0.2 °C
**$MgCl_2$**	-1.6 °C	-2.1°C	-0.9 °C	1.2°C
**KCl**	1.0 °C	1.2 °C	1.2 °C	1.8 °C
**NaCl**	0.8 °C	0.9 °C	1.1 °C	1.9 °C

Effect of different salts on melting temperature of GbpA and GbpA variants (comparison to sample without added salts, top row). For these experiments, the protein was in its apo form, not bound to copper. Measurements were performed in triplicates.

[a] Standard deviations are similar for the experiments with salts.

[b] $GbpA_{LPMO}$ refers to the isolated LPMO domain, whereas FL refers to full-length GbpA.

Table S3. Fitting of dissociation constants (K_d)

Single site ligand binding, stabilization for Ca^{2+}

**Best-fit values**	
Tm_{min} (°C)	56.5
Tm_{max} (°C)	59.2
P (protein concentration, mM)	= 0.0020
K_d (mM)	0.22
**95% CI (profile likelihood)**	
Tm_{min} (°C)	56.3 to 56.6
Tm_{max} (°C)	59.0 to 59.3
K_d (mM)	0.17 to 0.28
**Goodness of Fit**	
Degrees of Freedom	30
R squared	0.975
Sum of Squares	0.869
Sy.x	0.1701
**Constraints**	
P (protein concentration, mM)	P = 0.0020
**Number of points**	
# of X values	33
# Y values analyzed	33

Fitted equation

Y=Tm_{min} + ((Tm_{max} - Tm_{min})*(1-((P-K_d-X+sqrt(((P+X+K_d)^2)-(4*P*X)))/(2*P))))

Single site ligand binding, negative stabilization for Mg^{2+}

**Best-fit values**	
Tm_{min} (°C)	66.2
Tm_{max} (°C)	61.3
P (protein concentration, mM)	= 0.0020
K_d (mM)	2.2
**95% CI (profile likelihood)**	
Tm_{min} (°C)	66.1 to 66.3
Tm_{max} (°C)	61.1 to 61.6
K_d (mM)	1.9 to 2.7
**Goodness of Fit**	
Degrees of Freedom	33
R squared	0.985
Sum of Squares	1.618
Sy.x	0.2214
**Constraints**	
P (protein concentration, mM)	P = 0.0020
**Number of points**	
# of X values	36
# Y values analyzed	36

Fitted equation

Y=Tm_{max} - ((Tm_{max} - Tm_{min})*(1-((P-K_d-X+sqrt(((P+X+K_d)^2)-(4*P*X)))/(2*P))))

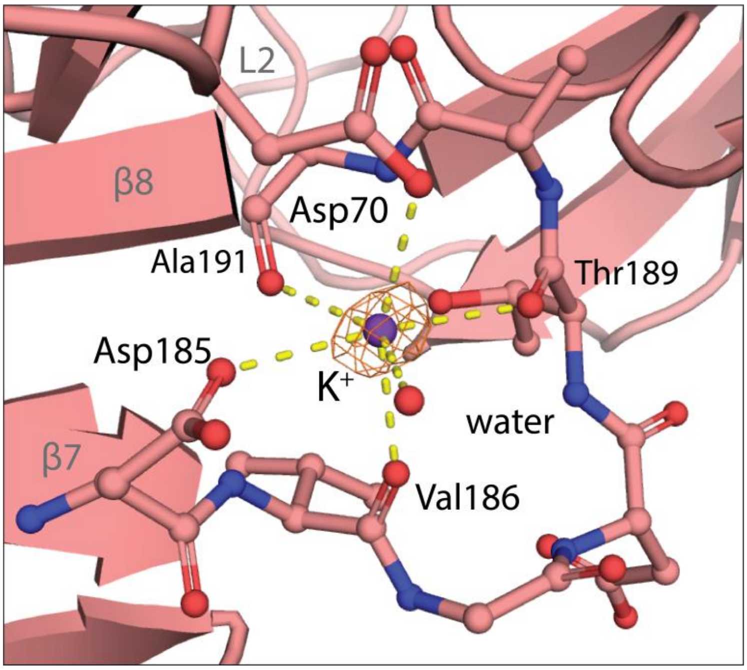

Figure S1. **Anomalous electron density of K⁺**. Close-up view of the cation-binding site featuring K^+ (purple sphere), with anomalous map contoured at 4σ (PDB ID: 7PB6; this work).

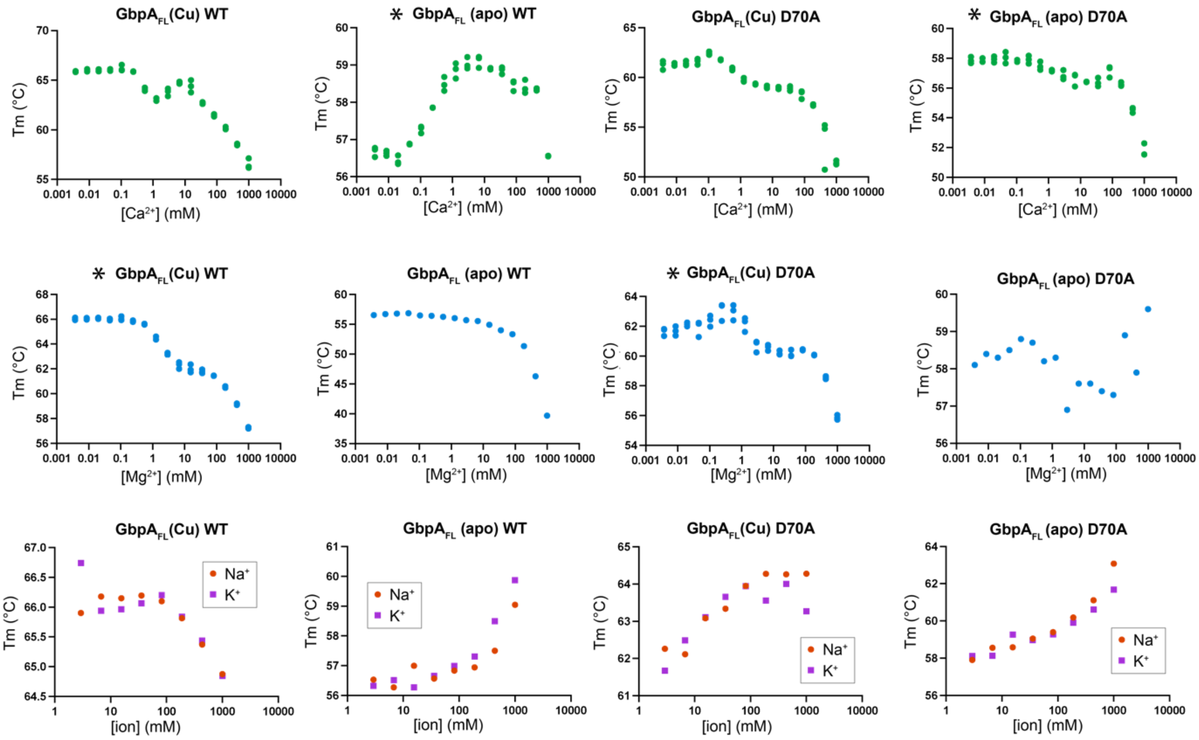

Figure S2. **Effect of different cations on GbpA stability**. Stability of $GbpA_{FL}$ subjected to different cations and ion concentrations. Experiments were performed for GbpA WT and D70A variants, both for apo (apo) and copper-saturated protein (Cu). Note the general destabilizing trend for divalent cations and stabilization by monovalent ions. Panels with a star * are also part of Figure 3.

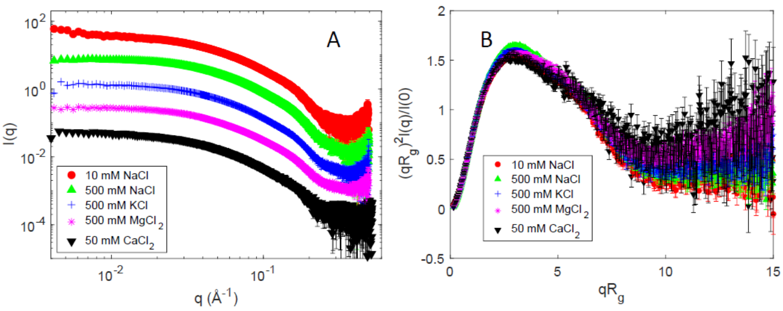

**Sample**	R_g **(Å)**
10 mM NaCl	38.7 +/- 0.6*
500 mM NaCl	39.0 +/- 0.2
500 mM KCl	38.7 +/- 0.7
500 mM $MgCl_2$	38.0 +/- 0.5
50 mM $CaCl_2$	39.6 +/- 0.6

Figure S3. **SAXS data for GbpA in the presence of different cations**. **A**. Intensities are plotted against the scattering vector q and arbitrarily scaled for visibility. The plots are merged from datasets of concentration series of GbpA exposed to the respective salts, by extrapolating to infinite dilution, hence eliminating concentration-dependent structure factor effects. The plots show limited salt effects on the overall structure of GbpA, but with slight aggregation in 10 mM NaCl, where the salt concentration might be insufficient to screen out inter-particle effects. All data were collected at pH 7.0. For $CaCl_2$, data were only collected at 50 mM, and a different buffer (HEPES) was used, since higher salt concentrations and different buffers led to radiation damage. **B**. Dimensionless Kratky plots show that GbpA is folded and globular under all conditions, as the plots are bell-shaped rather than having a consistent inclination at high qR_g, a characteristic of unfolded proteins. The 500 mM $MgCl_2$ and 50 mM $CaCl_2$ samples do not reach the same low plateau, but the difference is within the margin of error. In the table to the right, the R_g from Guinier approximation is shown for each sample. No significant difference in R_g is observed. *For 10 mM NaCl, data below q = 0.012 $Å^{-1}$ were omitted from the Guinier analysis.

				70				
2XWX	GbpA/VcAA10B	*Vibrio cholerae*	65	SVEGPDGFP--	73	180	ILAVWDVG---DTAASFYNV	196
8GUL	GbpA/VhAA10A	*Vibrio campbellii*	65	SVEGPDGFP--	73	180	ILAVWDVG---DTAASFYNV	196
2BEM	CBP21	*Serratia marcescens*	31	SVEGLKGFP--	39	147	ILAVWDIA---DTANAFYQA	163
6T5Z	CbpL	*Photorhabdus laumondii*	34	SLEAKKGFP--	42	150	ILGVWTIS---DTLNAFYQV	166
2YOW	BaAA10A	*Bacillus amyloliquefaciens*	39	SVEGPKGFP--	47	156	ILGVWDVA---DTSNAFYNV	172
5LW4	BlLPMO10A	*Bacillus liqueniformis*	34	SLEAKKGFP--	42	148	ILAVWDVA---DTENAFYQV	164
5L2V	LMRG_01781	*Listeria monocytogenes*	31	SVEAPKGFP--	39	143	ILGVWNIA---DTGNAFYQI	159
5FTZ	SlLPMO10E	*Streptomyces lividans*	31	SVEGPKGFP--	39	151	ILAVWTVH---DTGNAFYAC	167
4A02	EfCBM33A	*Enterococcus faecalis*	34	SIEAPKNTF--	42	144	IYAVWGIG---DTVNAFYQA	160
3UAM	BpAA10A	*Burkholderia pseudomallei*	47	ELEGGKFFPAT	57	194	LLAVWDVA---DTANAFYQV	210
5AA7	JdLPMO10A	*Jonesia denitrificans*	32	SVEAPKGAT--	40	120	ILARWNVS---NTNNAFYNC	136
5WSZ	BtLPMO10A	*Bacillus thuringiensis*	31	SVEGIGGFP--	39	145	ILAVWEIA---DTGNAFYQV	161
6RW7	TtAA10A	*Teredinibacter turnerae*	50	SVVAHHE----	56	181	IFAEWGRN--EHTYERFFSC	198
7OKR	AsLPMO10B	*Aliivibrio salmonicida*	72	EVAANVPNY--	80	194	LYTRWQRE--DAAGEGFYNC	211
5OPF	MaLPMO10B	*Micromonospora aurantica*	50	GLFREGV----	56	172	VYTIWQAS---HLDQSYYLC	188
5UIZ	TfLPMO10A	*Thermobifida fusca*	86	GLYRDWV----	92	200	VFTIWKAS---HMDQTYYLC	216
6NDQ	KpLPMO10A	*Kitasatospora papulosa*	50	GLYRNGS----	56	164	VYTIWQAS---HMDQTYFLC	180
4OY6	ScLPMO10B	*Streptomyces coelicolor*	50	GLYRNGS----	56	164	VYTIWQAS---HMDQTYFLC	180
5FJQ	CjLPMO10A	*Cellvibrio japonicus*	43	EVAVGGV----	49	155	IYSIWQRDWDRDAAEGFYQC	174
4OY7	ScLPMO10C	*Streptomyces coelicolor*	49	AVLDSNA----	55	172	IFMQWVRS---DSQENFFSC	188
7ZJB	SCO174	*Streptomyces coelicolor*	44	GVNQGNA----	50	163	IYNVWQRS---DSPEAFYAC	179
8C5N	CbpD	*Pseudomonas aeruginosa*	44	GIRIGNA----	50	163	LYAVWQRS---DSPEAFYSC	175
6IF7	Tma12	*Tectaria macrodonta*	44	EVNIPNA----	50	161	IYVIWQRT---DSPEAFYSC	177
4YN2	fusolin	unidentified entomopoxvirus	64	QDNEYAA----	70	208	LYVRWQRL--DPVGEGFYNC	225
4YN1	fusolin	*Anomala cuprea* antomopoxvirus CV6M	65	QDNEYAA----	71	211	IYVRWQRI--DPVGEGFYNC	228
4OW5	fusolin	*Melolontha melolontha* entomopoxvirus MMEV	65	QDNEYAA----	71	211	LYVRWQRN--DPVGEGFYNC	228

Figure S4. **Multiple sequence alignment of AA10 LPMOs**. Included in this structure-based alignment are all AA10 LPMO structures available in the PDB on 2/12/24 (with PDB IDs given on the left). Residue numbers are indicated for protein elements involved in cation-binding in GbpA, involving L2 loop residues and β-strands 7 to 8, with residues coordinating the metal ion highlighted. Asp70 (D in one-letter code; highlighted in green) is often replaced by Lys (K in one-letter code; highlighted in blue) in other AA10 LPMOs.

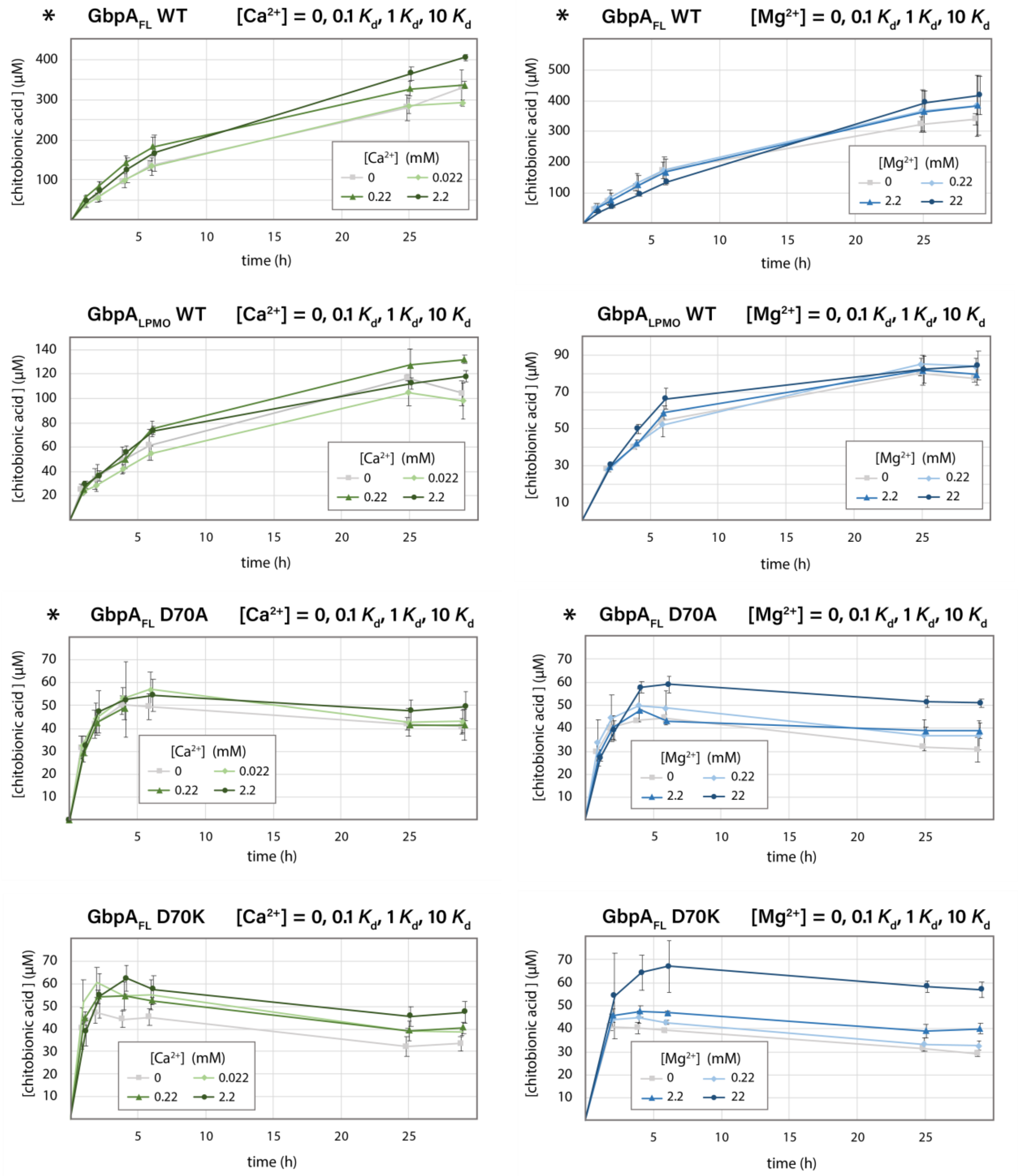

Figure S5. **Effect of salts on GbpA catalytic activity without externally added H_2O_2**. Experiments were performed for the full-length WT protein and variants D70A and D70K as well as for the isolated WT LPMO domain (GbpA$_{LPMO}$). Experiments labeled with a star * are also part of Figure 5. All experiments were performed in triplicates and the error bars refer to standard deviations.

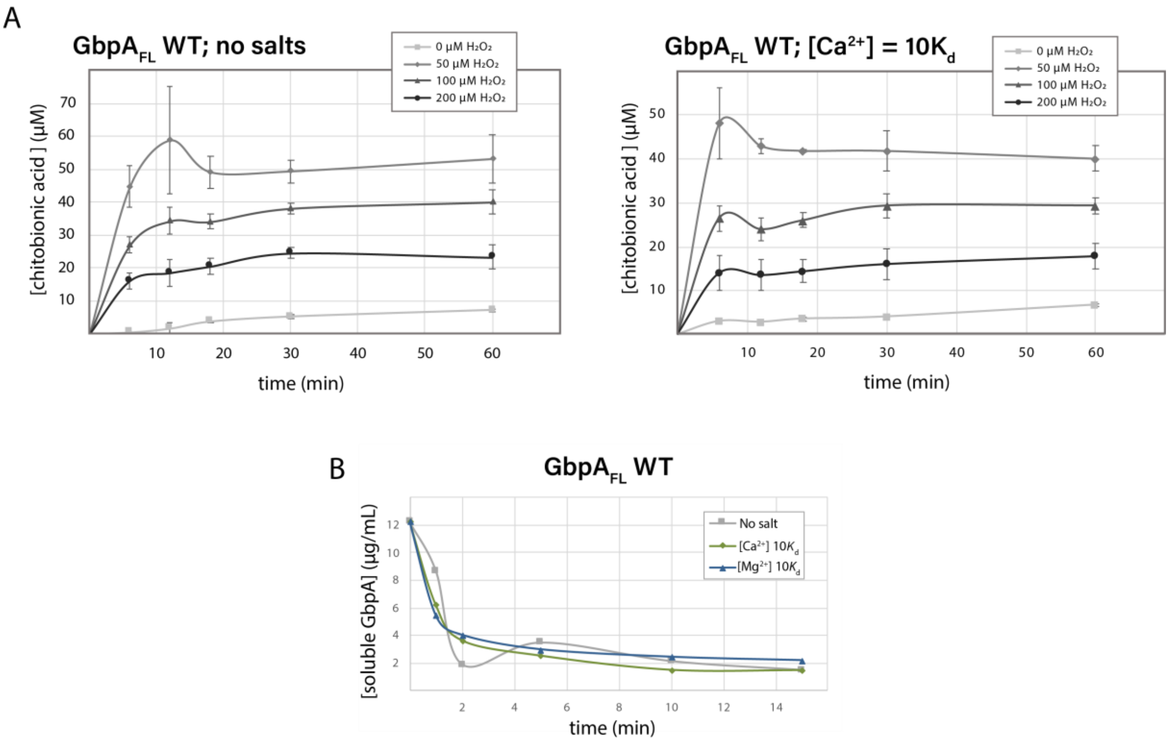

Figure S6. **Effect of salts on GbpA catalytic activity with H_2O_2 added as a co-substrate (A) and binding to chitin (B)**. **A.** The presence of calcium did not provide any protection against the oxidative damage usually assumed to be responsible for LPMO inactivation. Experiments were performed in triplicates and the error bars refer to standard deviations. **B.** Preliminary chitin-binding assays performed in the absence or presence of calcium and magnesium did not show significant differences worth exploring further. The binding equilibrium is reached after 10 min, suggesting that a 20 min preincubation with chitin is sufficient to load GbpA with chitin before starting the activity assays by adding ascorbic acid.

References

Harding MM (1999) The geometry of metal–ligand interactions relevant to proteins. *Acta Crystallographica Section D Biological Crystallography* **55**, 1432-1443.

Harding MM (2001) Geometry of metal–ligand interactions in proteins. *Acta Crystallographica Section D Biological Crystallography* **57**, 401-411.

Zheng H, Chruszcz M, Lasota P, Lebioda L and Minor W (2008) Data mining of metal ion environments present in protein structures. *Journal of Inorganic Biochemistry* **102**, 1765-1776.
