# Supplementary material for: Calcium-binding site in AA10 LPMO from Vibrio cholerae suggests modulating effects during environmental survival and infection
Source: QRB Discov. 2024 Dec 26;5:e12. doi: 10.1017/qrd.2024.14 (PMC11729483; doi:10.1017/qrd.2024.14)
Supplement: Peer review history

# Editor report: Decision: Calcium-binding site in AA10 LPMO from Vibrio cholerae suggests modulating effects during environmental survival and infection — R0/PR2

**DOI:** 10.1017/qrd.2024.14.pr2

**Affiliations:** Chemistry, Chalmers University of Technology, Sweden

No accompanying comment.

# Editor report: Recommendation: Calcium-binding site in AA10 LPMO from *Vibrio cholerae* suggests modulating effects during environmental survival and infection — R1/PR4

**DOI:** 10.1017/qrd.2024.14.pr4

**Affiliations:** Chemistry, Chalmers University of Technology, Sweden

No accompanying comment.

# Editor report: Decision: Calcium-binding site in AA10 LPMO from *Vibrio cholerae* suggests modulating effects during environmental survival and infection — R1/PR5

**DOI:** 10.1017/qrd.2024.14.pr5

**Affiliations:** Chemistry, Chalmers University of Technology, Sweden

No accompanying comment.
